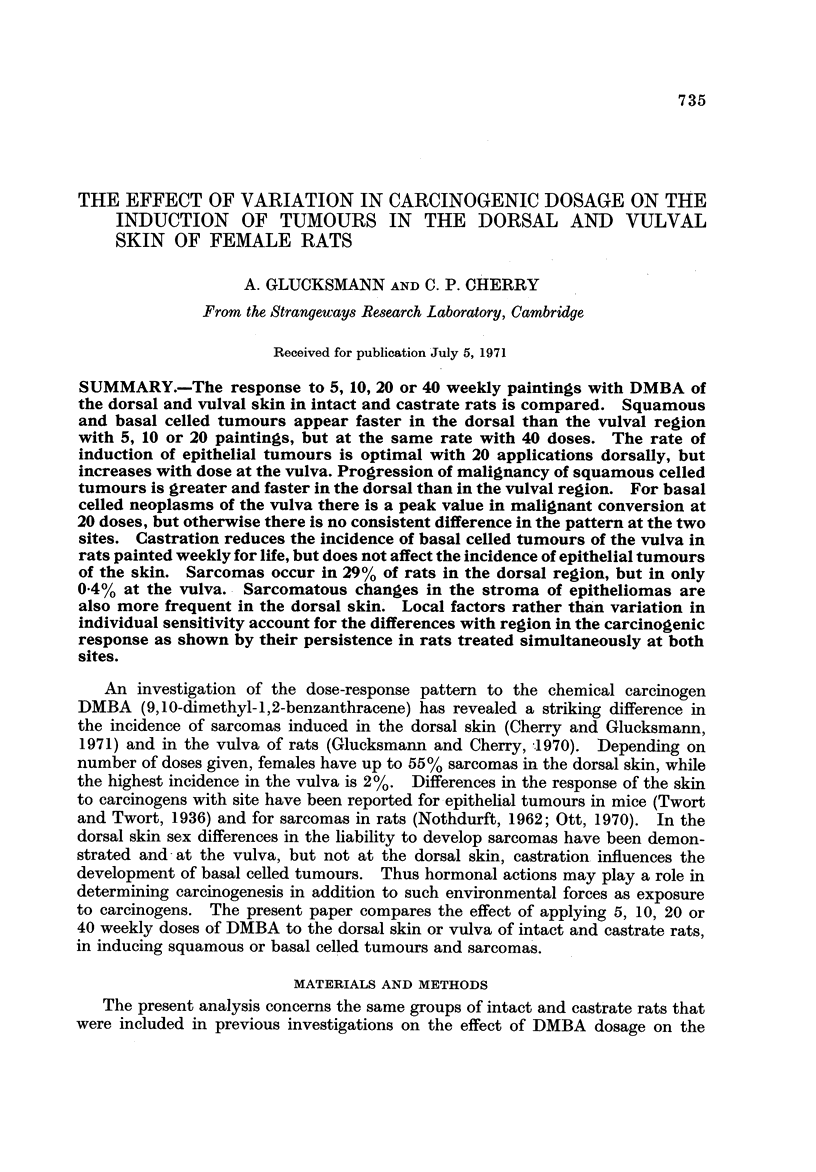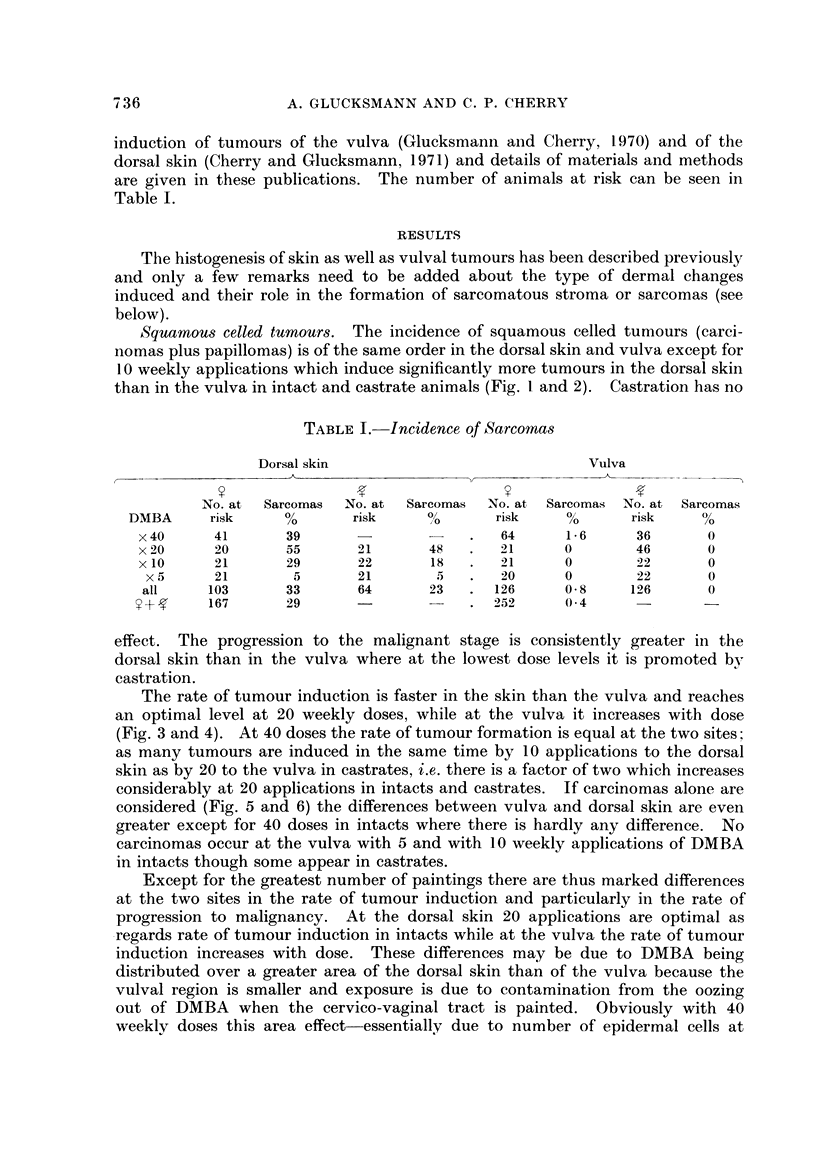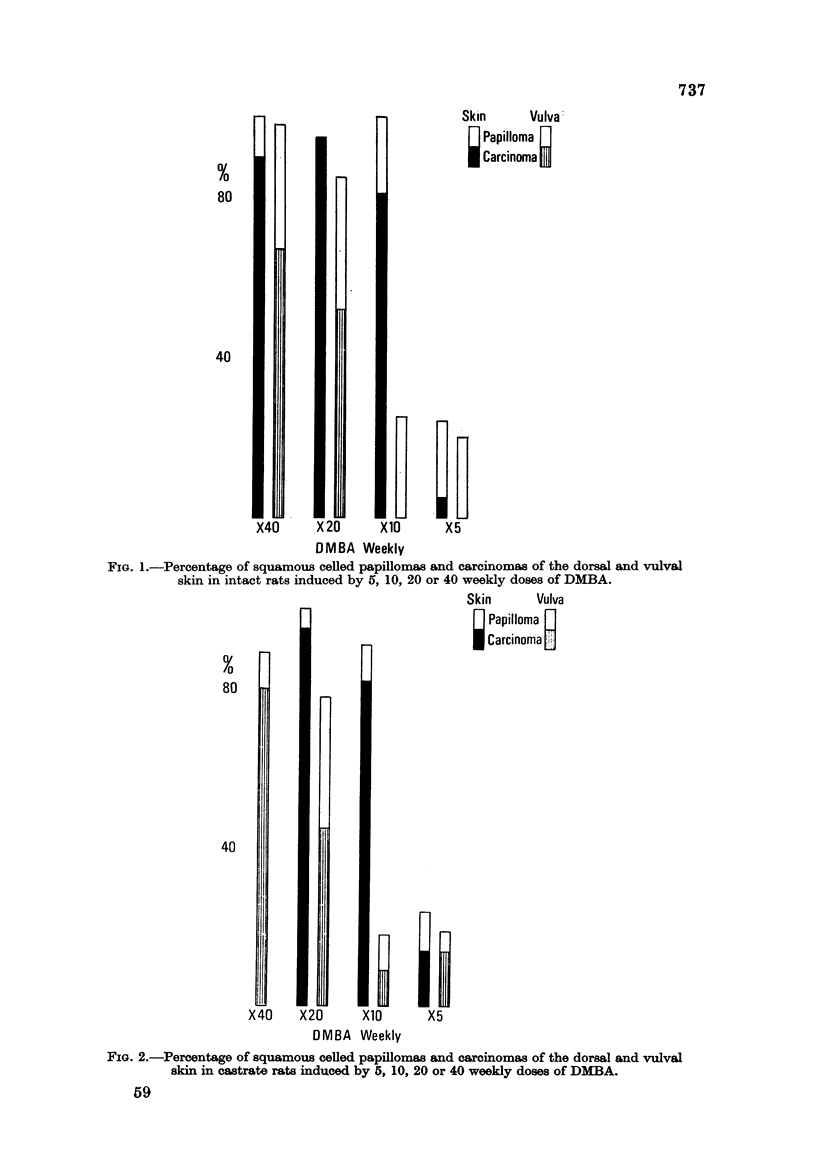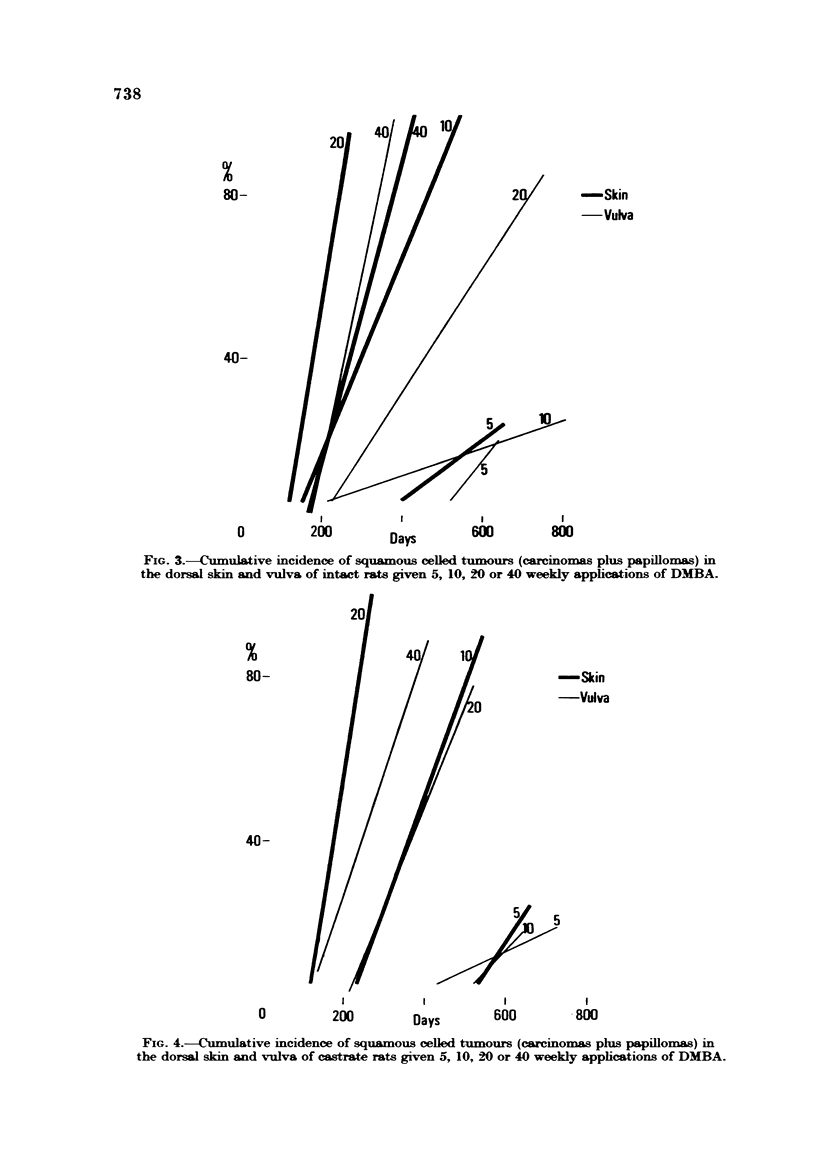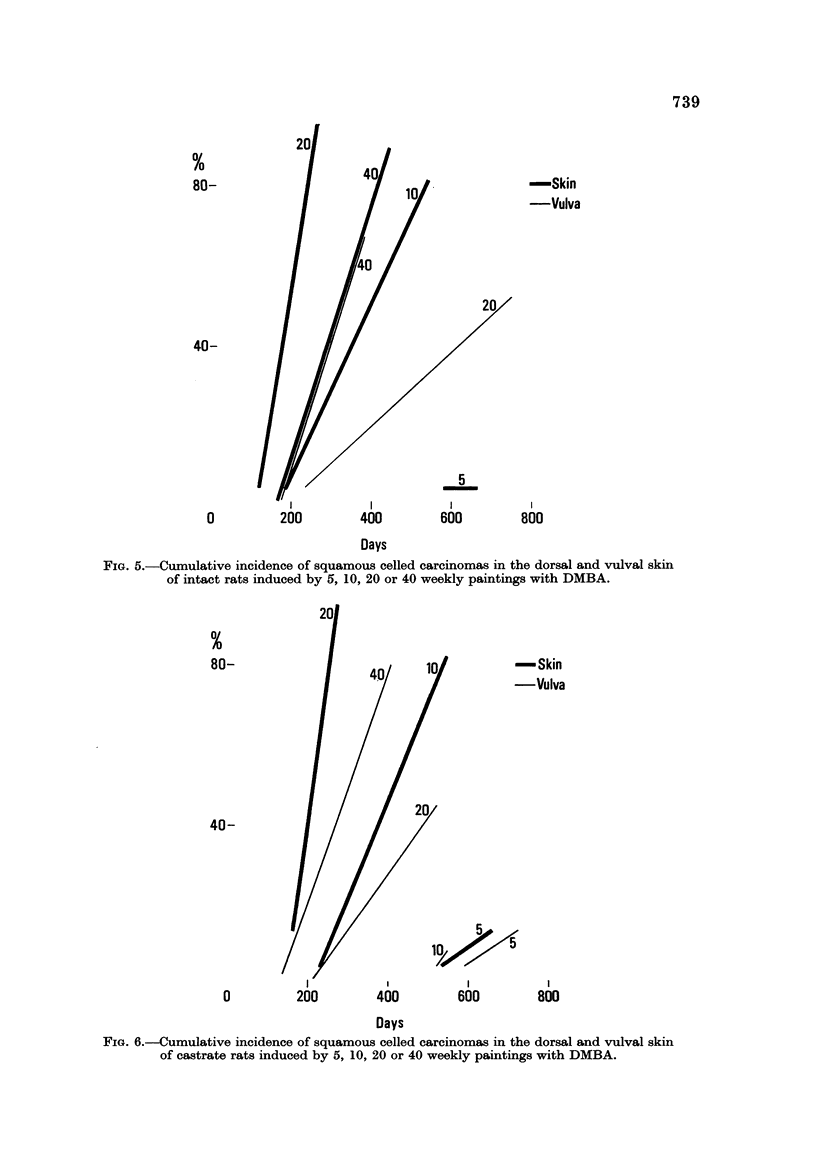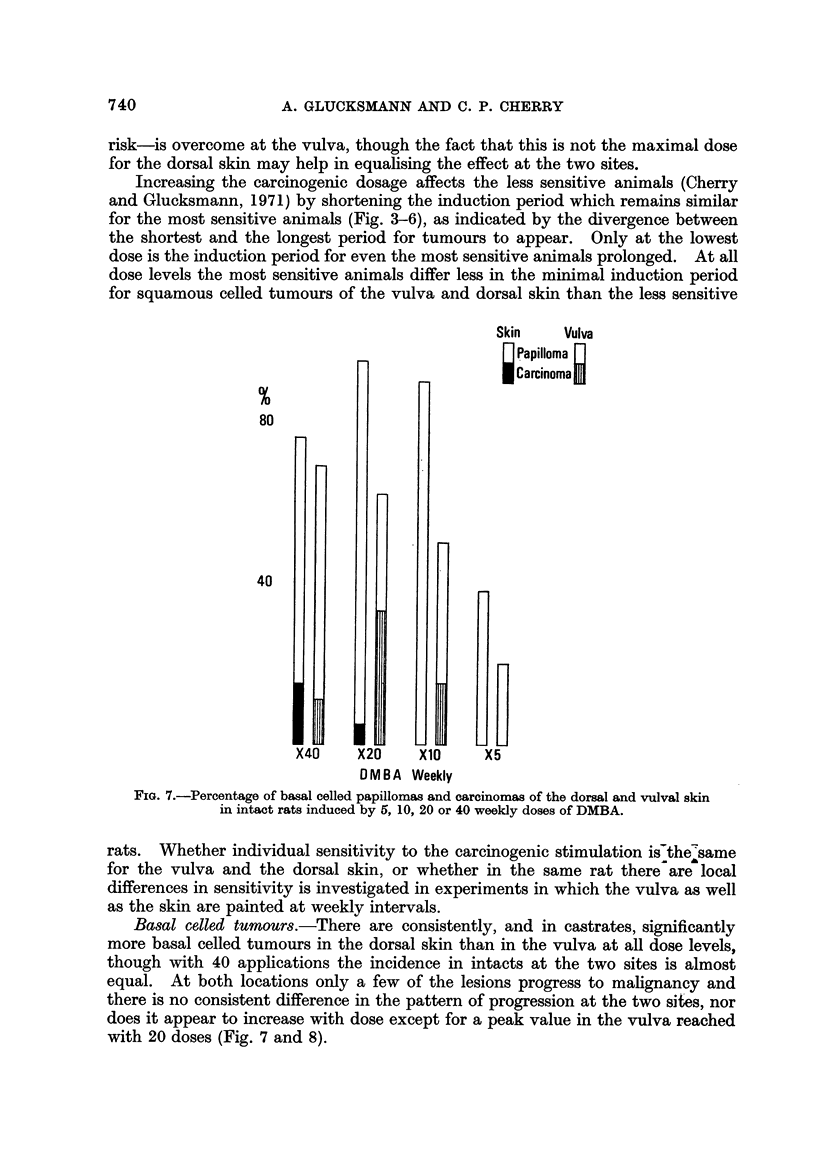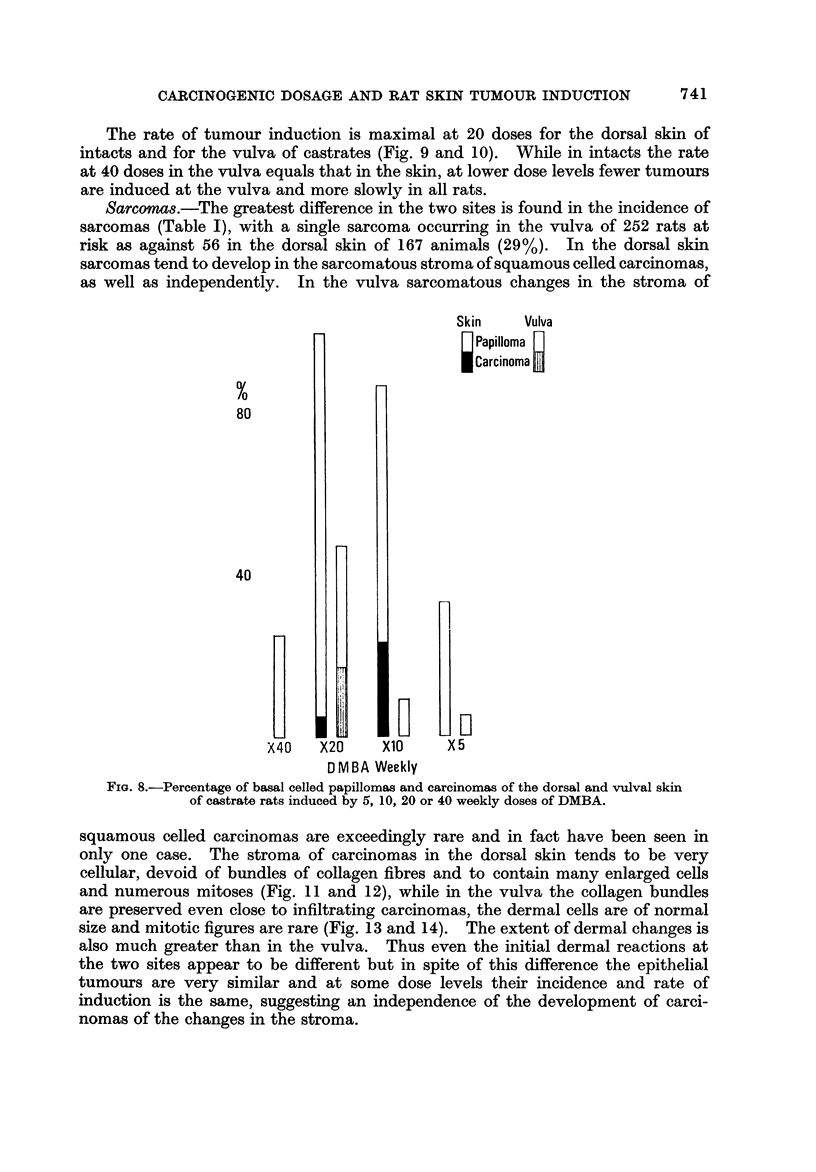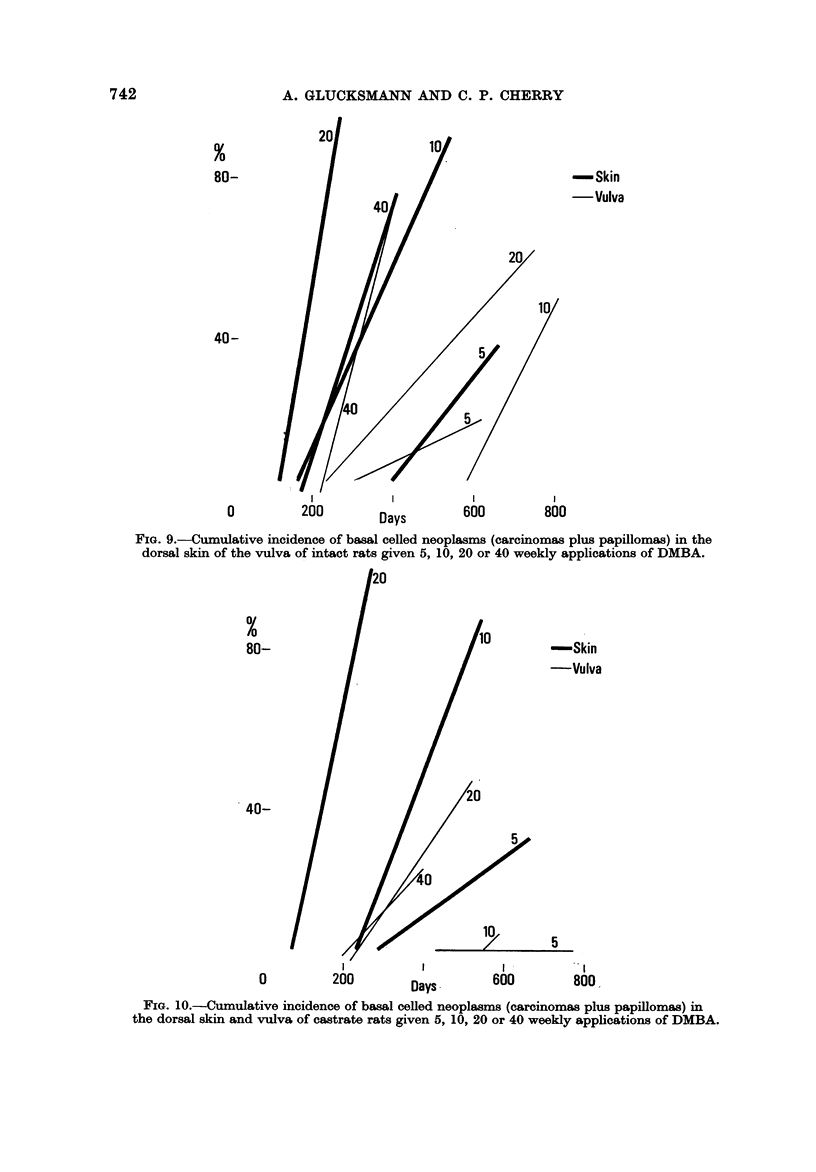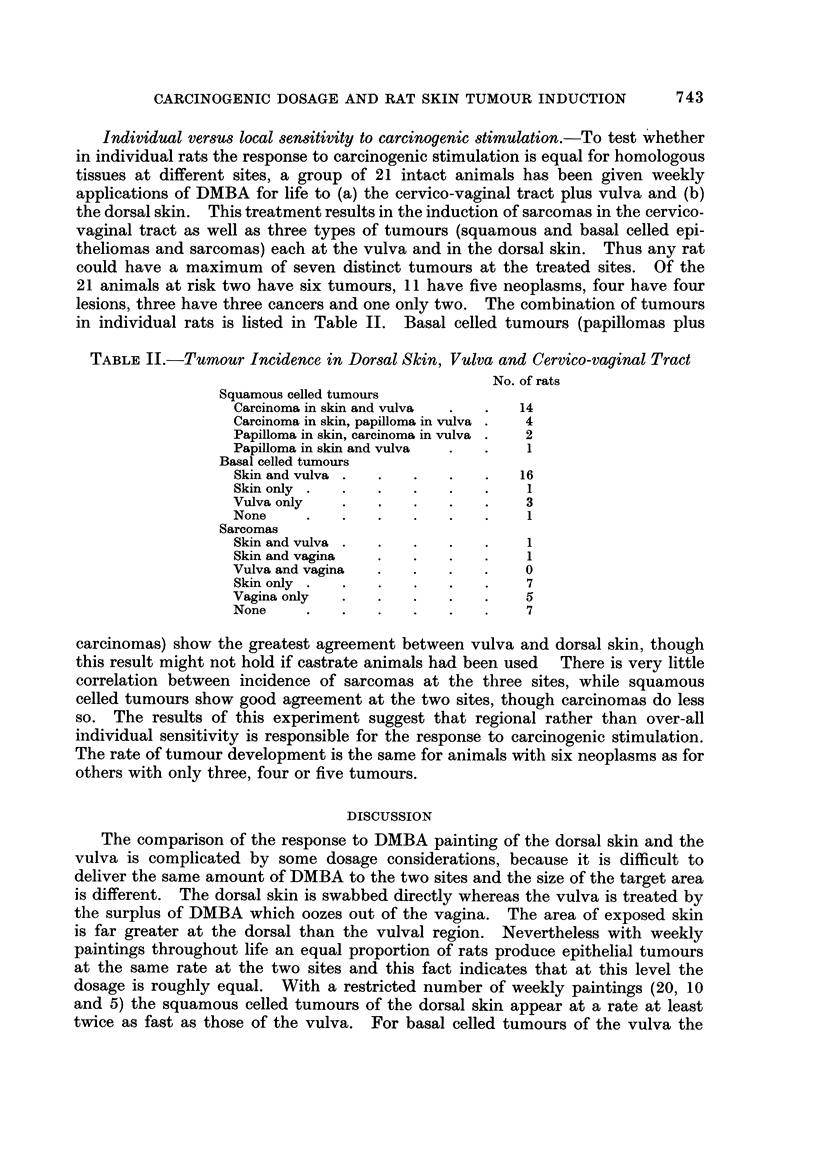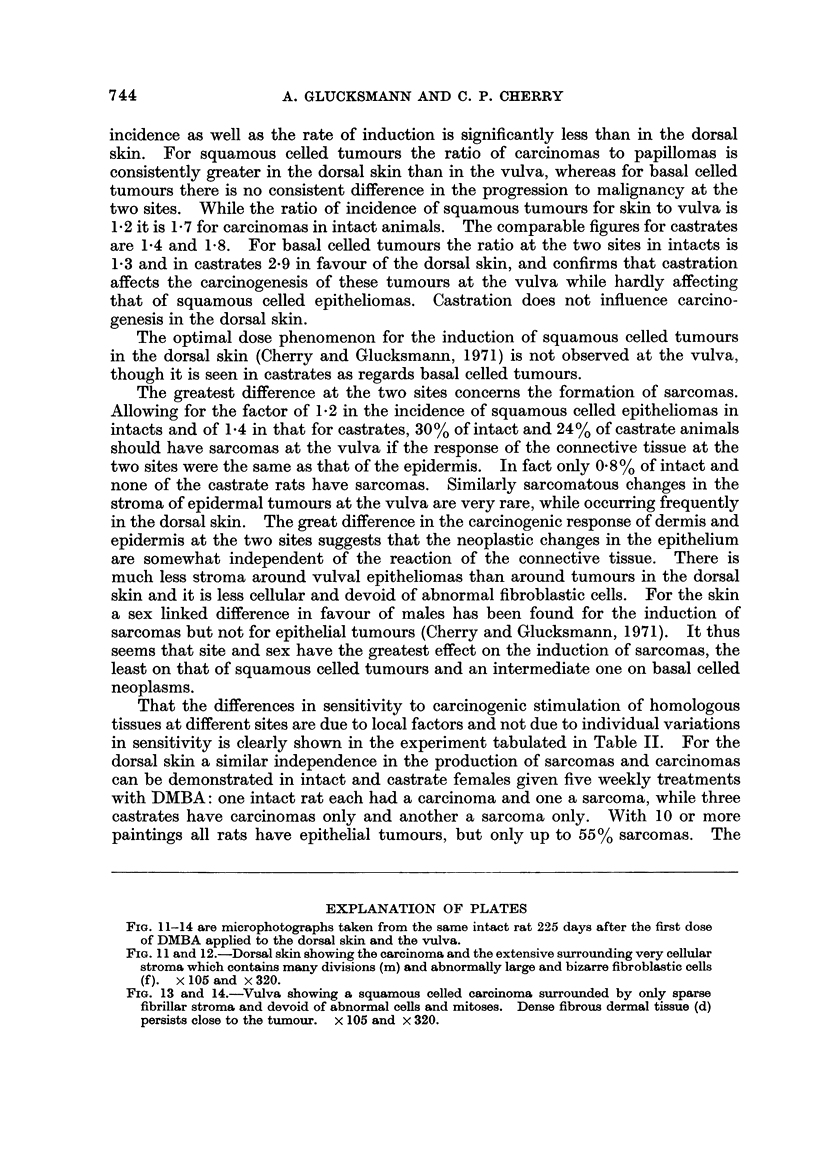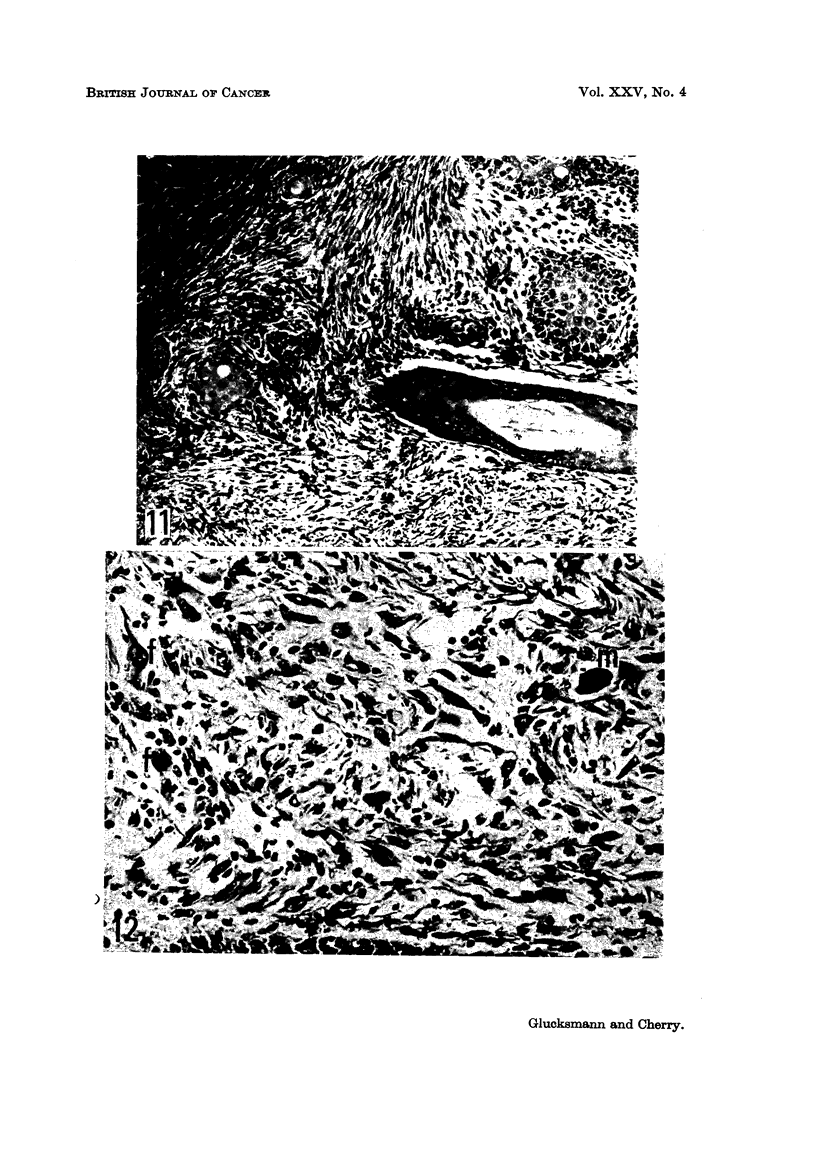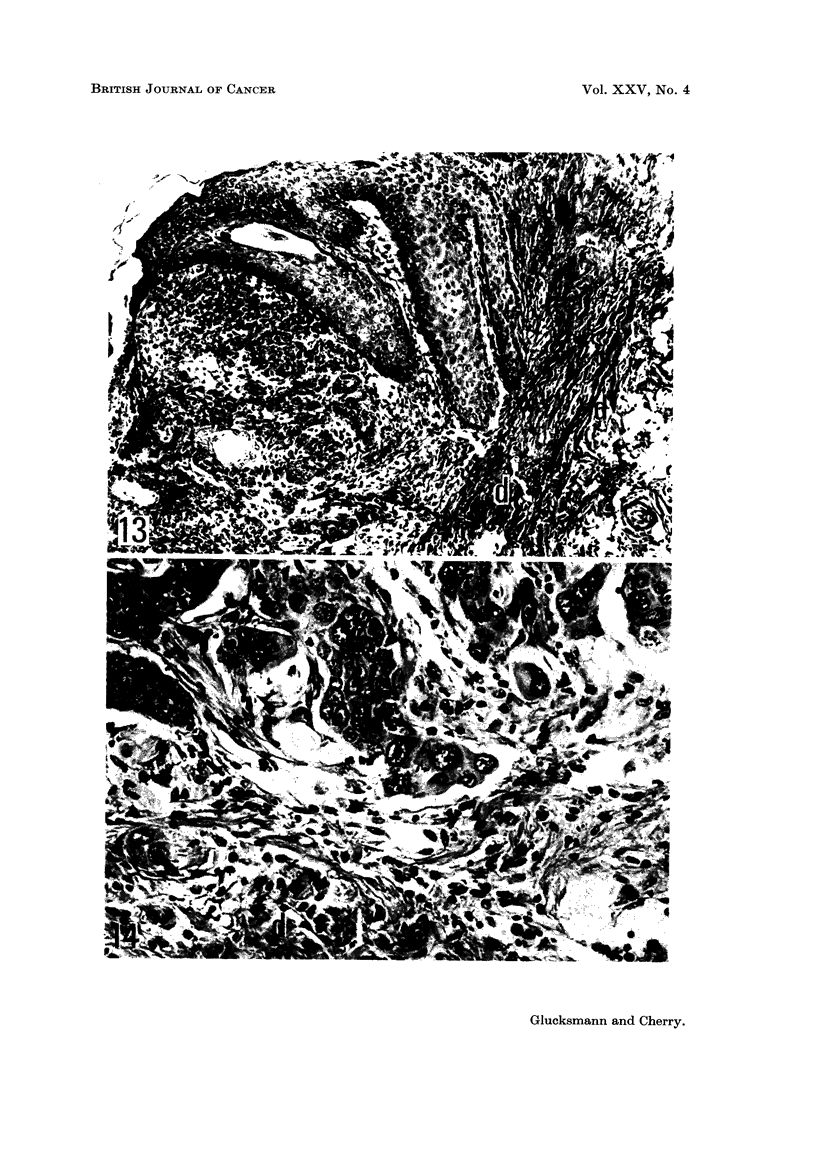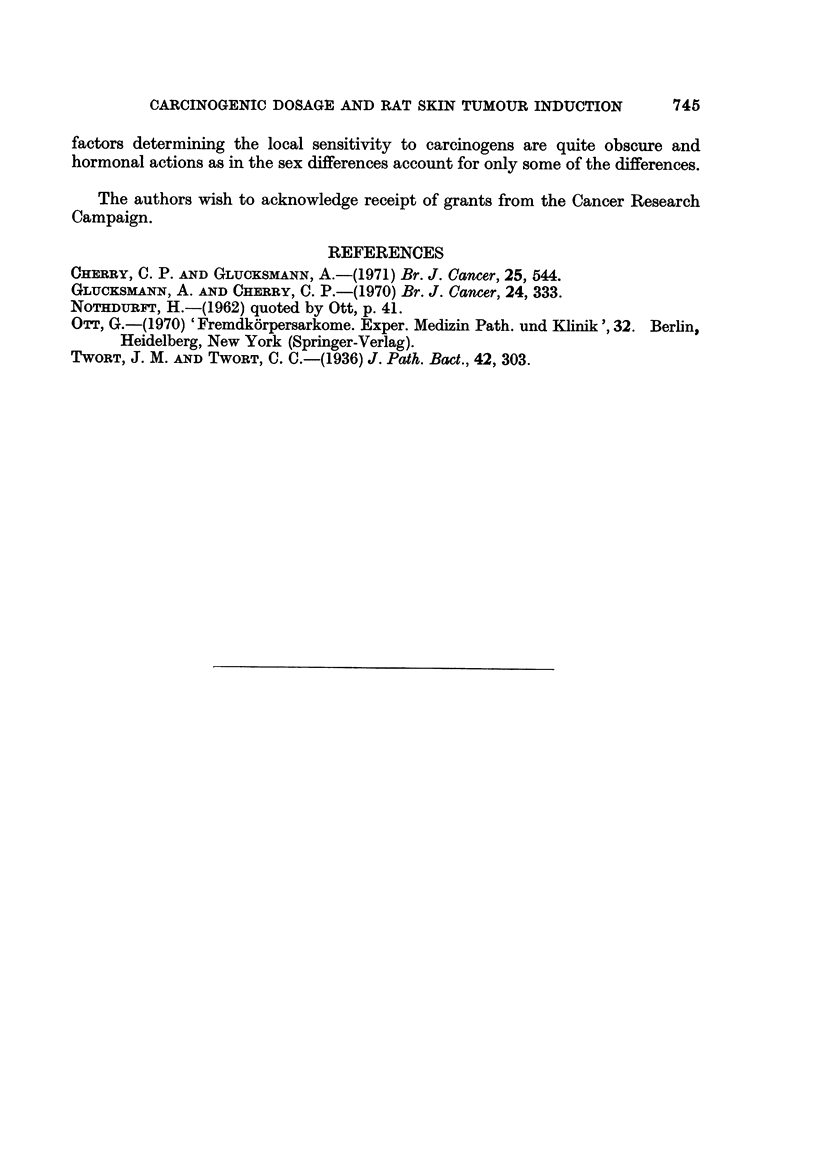# The Effect of Variation in Carcinogenic Dosage on the Induction of Tumours in the Dorsal and Vulval Skin of Female Rats

**DOI:** 10.1038/bjc.1971.88

**Published:** 1971-12

**Authors:** A. Glucksmann, C. P. Cherry

## Abstract

**Images:**


### THE EFFECT OF VARIATION IN CARCINOGENIC DOSAGE ON THE INDUCTION OF TUMOURS IN THE DORSAL AND VULVAL SKIN OF FEMALE RATS

A. GLUCKSMANN AND C. P. CHERRY

*From the Strangeways Research Laboratory, Cambridge*

Received for publication July 5, 1971

**SUMMARY.—The response to 5, 10, 20 or 40 weekly paintings with DMBA of the dorsal and vulval skin in intact and castrate rats is compared. Squamous and basal celled tumours appear faster in the dorsal than the vulval region with 5, 10 or 20 paintings, but at the same rate with 40 doses. The rate of induction of epithelial tumours is optimal with 20 applications dorsally, but increases with dose at the vulva. Progression of malignancy of squamous celled tumours is greater and faster in the dorsal than in the vulval region. For basal celled neoplasms of the vulva there is a peak value in malignant conversion at 20 doses, but otherwise there is no consistent difference in the pattern at the two sites. Castration reduces the incidence of basal celled tumours of the vulva in rats painted weekly for life, but does not affect the incidence of epithelial tumours of the skin. Sarcomas occur in 29% of rats in the dorsal region, but in only 0·4% at the vulva. Sarcomatous changes in the stroma of epitheliomas are also more frequent in the dorsal skin. Local factors rather than variation in individual sensitivity account for the differences with region in the carcinogenic response as shown by their persistence in rats treated simultaneously at both sites.**

An investigation of the dose-response pattern to the chemical carcinogen DMBA (9,10-dimethyl-1,2-benzanthracene) has revealed a striking difference in the incidence of sarcomas induced in the dorsal skin (Cherry and Glucksmann, 1971) and in the vulva of rats (Glucksmann and Cherry, 1970). Depending on number of doses given, females have up to 55% sarcomas in the dorsal skin, while the highest incidence in the vulva is 2%. Differences in the response of the skin to carcinogens with site have been reported for epithelial tumours in mice (Twort and Twort, 1936) and for sarcomas in rats (Nothdurft, 1962; Ott, 1970). In the dorsal skin sex differences in the liability to develop sarcomas have been demonstrated and at the vulva, but not at the dorsal skin, castration influences the development of basal celled tumours. Thus hormonal actions may play a role in determining carcinogenesis in addition to such environmental forces as exposure to carcinogens. The present paper compares the effect of applying 5, 10, 20 or 40 weekly doses of DMBA to the dorsal skin or vulva of intact and castrate rats, in inducing squamous or basal celled tumours and sarcomas.

### MATERIALS AND METHODS

The present analysis concerns the same groups of intact and castrate rats that were included in previous investigations on the effect of DMBA dosage on the induction of tumours of the vulva (Glucksmann and Cherry, 1970) and of the dorsal skin (Cherry and Glucksmann, 1971) and details of materials and methods are given in these publications. The number of animals at risk can be seen in Table I.

### RESULTS

The histogenesis of skin as well as vulval tumours has been described previously and only a few remarks need to be added about the type of dermal changes induced and their role in the formation of sarcomatous stroma or sarcomas (see below).

*Squamous celled tumours.* The incidence of squamous celled tumours (carcinomas plus papillomas) is of the same order in the dorsal skin and vulva except for 10 weekly applications which induce significantly more tumours in the dorsal skin than in the vulva in intact and castrate animals (Fig. 1 and 2). Castration has no

TABLE I.—*Incidence of Sarcomas*

| | Dorsal skin | | | | Vulva | | | |
|---|---|---|---|---|---|---|---|---|
| DMBA | ♀ No. at risk | Sarcomas % | ♀̸ No. at risk | Sarcomas % | ♀ No. at risk | Sarcomas % | ♀̸ No. at risk | Sarcomas % |
| ×40 | 41 | 39 | — | — | 64 | 1·6 | 36 | 0 |
| ×20 | 20 | 55 | 21 | 48 | 21 | 0 | 46 | 0 |
| ×10 | 21 | 29 | 22 | 18 | 21 | 0 | 22 | 0 |
| ×5 | 21 | 5 | 21 | 5 | 20 | 0 | 22 | 0 |
| all | 103 | 33 | 64 | 23 | 126 | 0·8 | 126 | 0 |
| ♀+♀̸ | 167 | 29 | — | — | 252 | 0·4 | — | — |

effect. The progression to the malignant stage is consistently greater in the dorsal skin than in the vulva where at the lowest dose levels it is promoted by castration.

The rate of tumour induction is faster in the skin than the vulva and reaches an optimal level at 20 weekly doses, while at the vulva it increases with dose (Fig. 3 and 4). At 40 doses the rate of tumour formation is equal at the two sites; as many tumours are induced in the same time by 10 applications to the dorsal skin as by 20 to the vulva in castrates, *i.e.* there is a factor of two which increases considerably at 20 applications in intacts and castrates. If carcinomas alone are considered (Fig. 5 and 6) the differences between vulva and dorsal skin are even greater except for 40 doses in intacts where there is hardly any difference. No carcinomas occur at the vulva with 5 and with 10 weekly applications of DMBA in intacts though some appear in castrates.

Except for the greatest number of paintings there are thus marked differences at the two sites in the rate of tumour induction and particularly in the rate of progression to malignancy. At the dorsal skin 20 applications are optimal as regards rate of tumour induction in intacts while at the vulva the rate of tumour induction increases with dose. These differences may be due to DMBA being distributed over a greater area of the dorsal skin than of the vulva because the vulval region is smaller and exposure is due to contamination from the oozing out of DMBA when the cervico-vaginal tract is painted. Obviously with 40 weekly doses this area effect—essentially due to number of epidermal cells at

FIG. 1.—Percentage of squamous celled papillomas and carcinomas of the dorsal and vulval skin in intact rats induced by 5, 10, 20 or 40 weekly doses of DMBA.

FIG. 2.—Percentage of squamous celled papillomas and carcinomas of the dorsal and vulval skin in castrate rats induced by 5, 10, 20 or 40 weekly doses of DMBA.

FIG. 3.—Cumulative incidence of squamous celled tumours (carcinomas plus papillomas) in the dorsal skin and vulva of intact rats given 5, 10, 20 or 40 weekly applications of DMBA.

FIG. 4.—Cumulative incidence of squamous celled tumours (carcinomas plus papillomas) in the dorsal skin and vulva of castrate rats given 5, 10, 20 or 40 weekly applications of DMBA.

FIG. 5.—Cumulative incidence of squamous celled carcinomas in the dorsal and vulval skin of intact rats induced by 5, 10, 20 or 40 weekly paintings with DMBA.

FIG. 6.—Cumulative incidence of squamous celled carcinomas in the dorsal and vulval skin of castrate rats induced by 5, 10, 20 or 40 weekly paintings with DMBA.

risk—is overcome at the vulva, though the fact that this is not the maximal dose for the dorsal skin may help in equalising the effect at the two sites.

Increasing the carcinogenic dosage affects the less sensitive animals (Cherry and Glucksmann, 1971) by shortening the induction period which remains similar for the most sensitive animals (Fig. 3–6), as indicated by the divergence between the shortest and the longest period for tumours to appear. Only at the lowest dose is the induction period for even the most sensitive animals prolonged. At all dose levels the most sensitive animals differ less in the minimal induction period for squamous celled tumours of the vulva and dorsal skin than the less sensitive

FIG. 7.—Percentage of basal celled papillomas and carcinomas of the dorsal and vulval skin in intact rats induced by 5, 10, 20 or 40 weekly doses of DMBA.

rats. Whether individual sensitivity to the carcinogenic stimulation is the same for the vulva and the dorsal skin, or whether in the same rat there are local differences in sensitivity is investigated in experiments in which the vulva as well as the skin are painted at weekly intervals.

*Basal celled tumours.*—There are consistently, and in castrates, significantly more basal celled tumours in the dorsal skin than in the vulva at all dose levels, though with 40 applications the incidence in intacts at the two sites is almost equal. At both locations only a few of the lesions progress to malignancy and there is no consistent difference in the pattern of progression at the two sites, nor does it appear to increase with dose except for a peak value in the vulva reached with 20 doses (Fig. 7 and 8).

The rate of tumour induction is maximal at 20 doses for the dorsal skin of intacts and for the vulva of castrates (Fig. 9 and 10). While in intacts the rate at 40 doses in the vulva equals that in the skin, at lower dose levels fewer tumours are induced at the vulva and more slowly in all rats.

*Sarcomas.*—The greatest difference in the two sites is found in the incidence of sarcomas (Table I), with a single sarcoma occurring in the vulva of 252 rats at risk as against 56 in the dorsal skin of 167 animals (29%). In the dorsal skin sarcomas tend to develop in the sarcomatous stroma of squamous celled carcinomas, as well as independently. In the vulva sarcomatous changes in the stroma of

FIG. 8.—Percentage of basal celled papillomas and carcinomas of the dorsal and vulval skin of castrate rats induced by 5, 10, 20 or 40 weekly doses of DMBA.

squamous celled carcinomas are exceedingly rare and in fact have been seen in only one case. The stroma of carcinomas in the dorsal skin tends to be very cellular, devoid of bundles of collagen fibres and to contain many enlarged cells and numerous mitoses (Fig. 11 and 12), while in the vulva the collagen bundles are preserved even close to infiltrating carcinomas, the dermal cells are of normal size and mitotic figures are rare (Fig. 13 and 14). The extent of dermal changes is also much greater than in the vulva. Thus even the initial dermal reactions at the two sites appear to be different but in spite of this difference the epithelial tumours are very similar and at some dose levels their incidence and rate of induction is the same, suggesting an independence of the development of carcinomas of the changes in the stroma.

FIG. 9.—Cumulative incidence of basal celled neoplasms (carcinomas plus papillomas) in the dorsal skin of the vulva of intact rats given 5, 10, 20 or 40 weekly applications of DMBA.

FIG. 10.—Cumulative incidence of basal celled neoplasms (carcinomas plus papillomas) in the dorsal skin and vulva of castrate rats given 5, 10, 20 or 40 weekly applications of DMBA.

*Individual versus local sensitivity to carcinogenic stimulation.*—To test whether in individual rats the response to carcinogenic stimulation is equal for homologous tissues at different sites, a group of 21 intact animals has been given weekly applications of DMBA for life to (a) the cervico-vaginal tract plus vulva and (b) the dorsal skin. This treatment results in the induction of sarcomas in the cervico-vaginal tract as well as three types of tumours (squamous and basal celled epitheliomas and sarcomas) each at the vulva and in the dorsal skin. Thus any rat could have a maximum of seven distinct tumours at the treated sites. Of the 21 animals at risk two have six tumours, 11 have five neoplasms, four have four lesions, three have three cancers and one only two. The combination of tumours in individual rats is listed in Table II. Basal celled tumours (papillomas plus carcinomas) show the greatest agreement between vulva and dorsal skin, though this result might not hold if castrate animals had been used There is very little correlation between incidence of sarcomas at the three sites, while squamous celled tumours show good agreement at the two sites, though carcinomas do less so. The results of this experiment suggest that regional rather than over-all individual sensitivity is responsible for the response to carcinogenic stimulation. The rate of tumour development is the same for animals with six neoplasms as for others with only three, four or five tumours.

TABLE II.—*Tumour Incidence in Dorsal Skin, Vulva and Cervico-vaginal Tract*

| | No. of rats |
|---|---|
| Squamous celled tumours | |
| Carcinoma in skin and vulva | 14 |
| Carcinoma in skin, papilloma in vulva | 4 |
| Papilloma in skin, carcinoma in vulva | 2 |
| Papilloma in skin and vulva | 1 |
| Basal celled tumours | |
| Skin and vulva | 16 |
| Skin only | 1 |
| Vulva only | 3 |
| None | 1 |
| Sarcomas | |
| Skin and vulva | 1 |
| Skin and vagina | 1 |
| Vulva and vagina | 0 |
| Skin only | 7 |
| Vagina only | 5 |
| None | 7 |

### DISCUSSION

The comparison of the response to DMBA painting of the dorsal skin and the vulva is complicated by some dosage considerations, because it is difficult to deliver the same amount of DMBA to the two sites and the size of the target area is different. The dorsal skin is swabbed directly whereas the vulva is treated by the surplus of DMBA which oozes out of the vagina. The area of exposed skin is far greater at the dorsal than the vulval region. Nevertheless with weekly paintings throughout life an equal proportion of rats produce epithelial tumours at the same rate at the two sites and this fact indicates that at this level the dosage is roughly equal. With a restricted number of weekly paintings (20, 10 and 5) the squamous celled tumours of the dorsal skin appear at a rate at least twice as fast as those of the vulva. For basal celled tumours of the vulva the incidence as well as the rate of induction is significantly less than in the dorsal skin. For squamous celled tumours the ratio of carcinomas to papillomas is consistently greater in the dorsal skin than in the vulva, whereas for basal celled tumours there is no consistent difference in the progression to malignancy at the two sites. While the ratio of incidence of squamous tumours for skin to vulva is 1·2 it is 1·7 for carcinomas in intact animals. The comparable figures for castrates are 1·4 and 1·8. For basal celled tumours the ratio at the two sites in intacts is 1·3 and in castrates 2·9 in favour of the dorsal skin, and confirms that castration affects the carcinogenesis of these tumours at the vulva while hardly affecting that of squamous celled epitheliomas. Castration does not influence carcinogenesis in the dorsal skin.

The optimal dose phenomenon for the induction of squamous celled tumours in the dorsal skin (Cherry and Glucksmann, 1971) is not observed at the vulva, though it is seen in castrates as regards basal celled tumours.

The greatest difference at the two sites concerns the formation of sarcomas. Allowing for the factor of 1·2 in the incidence of squamous celled epitheliomas in intacts and of 1·4 in that for castrates, 30% of intact and 24% of castrate animals should have sarcomas at the vulva if the response of the connective tissue at the two sites were the same as that of the epidermis. In fact only 0·8% of intact and none of the castrate rats have sarcomas. Similarly sarcomatous changes in the stroma of epidermal tumours at the vulva are very rare, while occurring frequently in the dorsal skin. The great difference in the carcinogenic response of dermis and epidermis at the two sites suggests that the neoplastic changes in the epithelium are somewhat independent of the reaction of the connective tissue. There is much less stroma around vulval epitheliomas than around tumours in the dorsal skin and it is less cellular and devoid of abnormal fibroblastic cells. For the skin a sex linked difference in favour of males has been found for the induction of sarcomas but not for epithelial tumours (Cherry and Glucksmann, 1971). It thus seems that site and sex have the greatest effect on the induction of sarcomas, the least on that of squamous celled tumours and an intermediate one on basal celled neoplasms.

That the differences in sensitivity to carcinogenic stimulation of homologous tissues at different sites are due to local factors and not due to individual variations in sensitivity is clearly shown in the experiment tabulated in Table II. For the dorsal skin a similar independence in the production of sarcomas and carcinomas can be demonstrated in intact and castrate females given five weekly treatments with DMBA: one intact rat each had a carcinoma and one a sarcoma, while three castrates have carcinomas only and another a sarcoma only. With 10 or more paintings all rats have epithelial tumours, but only up to 55% sarcomas. The

---

EXPLANATION OF PLATES

FIG. 11–14 are microphotographs taken from the same intact rat 225 days after the first dose of DMBA applied to the dorsal skin and the vulva.

FIG. 11 and 12.—Dorsal skin showing the carcinoma and the extensive surrounding very cellular stroma which contains many divisions (m) and abnormally large and bizarre fibroblastic cells (f). ×105 and ×320.

FIG. 13 and 14.—Vulva showing a squamous celled carcinoma surrounded by only sparse fibrillar stroma and devoid of abnormal cells and mitoses. Dense fibrous dermal tissue (d) persists close to the tumour. ×105 and ×320.

11

f
m
f
12

Glucksmann and Cherry.

factors determining the local sensitivity to carcinogens are quite obscure and hormonal actions as in the sex differences account for only some of the differences.

The authors wish to acknowledge receipt of grants from the Cancer Research Campaign.

### REFERENCES

CHERRY, C. P. AND GLUCKSMANN, A.—(1971) *Br. J. Cancer*, **25**, 544.
GLUCKSMANN, A. AND CHERRY, C. P.—(1970) *Br. J. Cancer*, **24**, 333.
NOTHDURFT, H.—(1962) quoted by Ott, p. 41.
OTT, G.—(1970) 'Fremdkörpersarkome. Exper. Medizin Path. und Klinik', **32**. Berlin, Heidelberg, New York (Springer-Verlag).
TWORT, J. M. AND TWORT, C. C.—(1936) *J. Path. Bact.*, **42**, 303.